# Placental Responses to Changes in the Maternal Environment Determine Fetal Growth

**DOI:** 10.3389/fphys.2016.00012

**Published:** 2016-01-29

**Authors:** Kris Genelyn Dimasuay, Philippe Boeuf, Theresa L. Powell, Thomas Jansson

**Affiliations:** ^1^Department of Medicine, The University of MelbourneMelbourne, VIC, Australia; ^2^Centre for Biomedical Research, Burnet InstituteMelbourne, VIC, Australia; ^3^Department of Obstetrics and Gynecology, University of Colorado Anschutz Medical CampusAurora, CO, USA; ^4^Victorian Infectious Diseases Service, Royal Melbourne HospitalMelbourne, VIC, Australia; ^5^Department of Pediatrics, University of Colorado Anschutz Medical CampusAurora, CO, USA

**Keywords:** placental nutrient sensing, maternal–fetal exchange, mechanistic target of rapamycin, fetal programming, syncytiotrophoblast, pregnancy

## Abstract

Placental responses to maternal perturbations are complex and remain poorly understood. Altered maternal environment during pregnancy such as hypoxia, stress, obesity, diabetes, toxins, altered nutrition, inflammation, and reduced *utero*-placental blood flow may influence fetal development, which can predispose to diseases later in life. The placenta being a metabolically active tissue responds to these perturbations by regulating the fetal supply of nutrients and oxygen and secretion of hormones into the maternal and fetal circulation. We have proposed that placental nutrient sensing integrates maternal and fetal nutritional cues with information from intrinsic nutrient sensing signaling pathways to balance fetal demand with the ability of the mother to support pregnancy by regulating maternal physiology, placental growth, and placental nutrient transport. Emerging evidence suggests that the nutrient-sensing signaling pathway mechanistic target of rapamycin (mTOR) plays a central role in this process. Thus, placental nutrient sensing plays a critical role in modulating maternal–fetal resource allocation, thereby affecting fetal growth and the life-long health of the fetus.

## Introduction

Adverse maternal influences in pregnancy are linked to alterations in the intrauterine milieu, which are associated with short-term complications including altered fetal growth and increased perinatal morbidity, as well as long-term adverse consequences for the health of the offspring. This concept of fetal programming or developmental origins of health and disease (Forsdahl, 1977; Barker and Osmond, 1986; Barker et al., 1989; Hales et al., 1991; Ravelli et al., 1998; Armitage et al., 2004; Gluckman and Hanson, 2004) suggests that successful prevention of adult metabolic disease relies on interventions during pregnancy.

The placenta senses and responds to changes in the maternal environment by altering its structure and function, which can lead to changes in blood flow, fetal nutrient supply, and secretion of hormones and other signaling molecules. Changes in transplacental nutrient transport may influence fetal nutrient availability, which determines fetal growth and body composition, and thus may link maternal perturbations to fetal programming.

The mechanisms by which altered maternal environment during pregnancy may lead to disease in the offspring are poorly understood. Here, we discuss maternal circulating factors that regulate placental function and highlight the role of placental mechanistic target of rapamycin (mTOR) signaling as a placental nutrient sensing signaling pathway that modifies placental nutrient transport in response to a multitude of factors.

## Placental nutrient sensing

**Figure 1:**
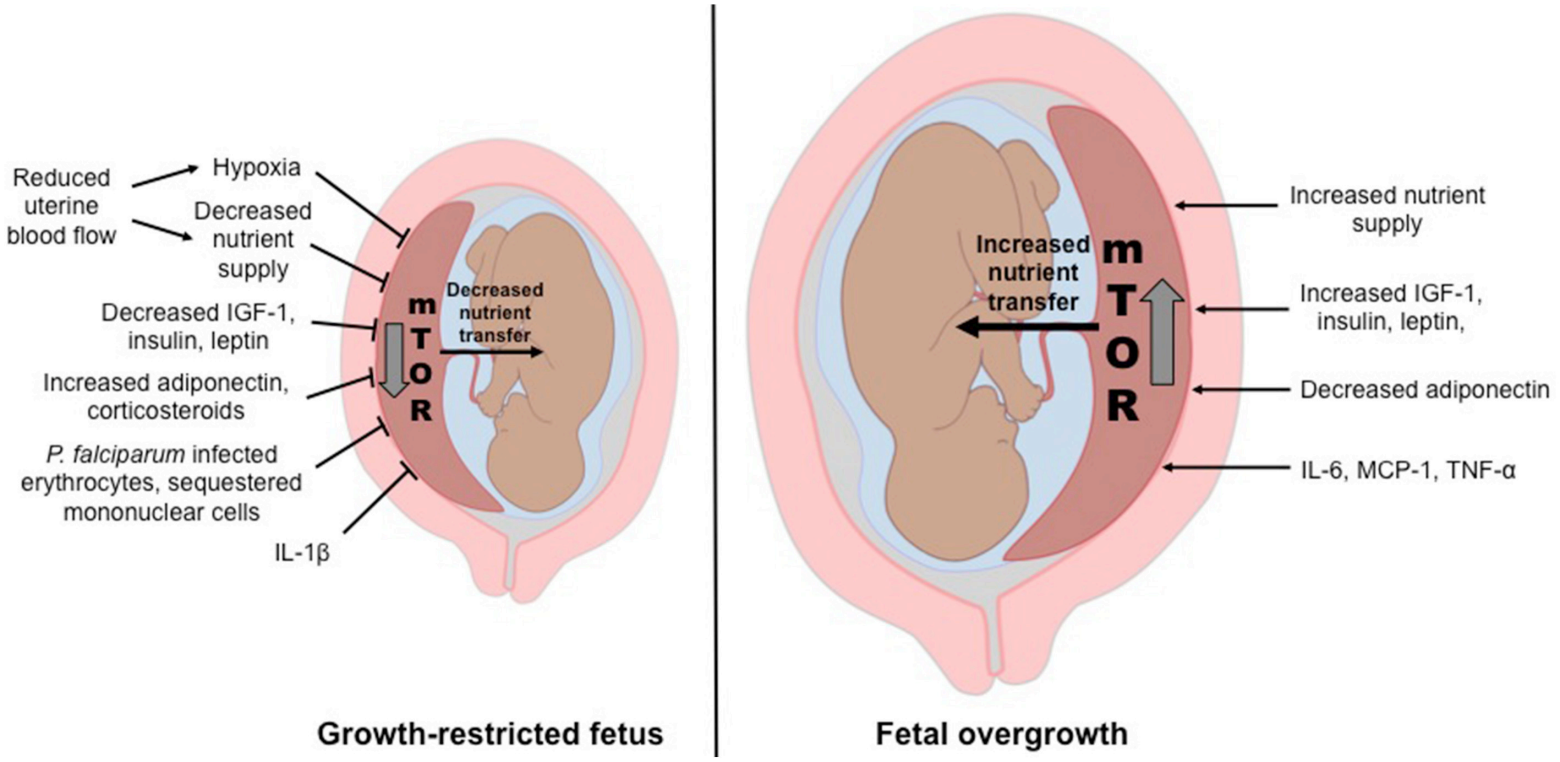
**Placental nutrient sensing model**. The placenta integrates maternal signals with information from nutrient sensing signaling pathways such as mechanistic target of rapamycin (mTOR) to regulate placental function. As a result, fetal growth is matched to the ability of the maternal supply line to allocate resources to the fetus. Thus, the placental responses to maternal signals influence fetal growth and the long-term health of the offspring.

The *placental nutrient sensing* model proposes that the syncytiotrophoblast integrates maternal and fetal signals to regulate placental function. The model emphasizes the importance of changes in the maternal compartment (Gaccioli et al., 2013; Jansson and Powell, 2013) to which the placenta responds by matching fetal growth with the ability of the maternal supply line to allocate resources to the fetus. Maternal signals that provide information to the placenta may include metabolic hormones, nutrients levels, and oxygen. In conditions of compromised ability of the maternal supply line to deliver nutrients and oxygen to the placenta, placental functions including transplacental nutrient transport and placental growth, may be inhibited, directly contributing to decreased fetal growth. In contrast, in conditions of over-nutrition, placental nutrient sensing may lead to enhanced placental function, directly contributing to fetal overgrowth (Figure 1).

## The placental barrier

The syncytiotrophoblast, the transporting and hormone-producing epithelium of the placenta, constitutes the primary barrier for maternal–fetal exchange. The syncytiotrophoblast is a polarized epithelium with a maternal-facing microvillous plasma membrane (MVM) and a fetal-facing basal plasma membrane (BM). MVM and BM have distinct biological characteristics including different membrane composition and their expression of nutrient transporters. The expression and function of these nutrient transporters influence the placental capacity to transfer nutrients from the mother to the fetus, an important determinant of fetal growth.

Maternal factors including hormones, growth factors, and some cytokines have been shown to regulate transplacental nutrient transport. Insulin (Jansson et al., 2003; Roos et al., 2009), insulin-like growth factor I (IGF-I; Fang et al., 1997; Roos et al., 2009), leptin (Jansson et al., 2003), interleukin-6 (IL-6; Jones et al., 2009a), and tumor necrosis factor alpha (TNF-α; Jones et al., 2007) positively regulate system A, a transport system that mediates non-essential neutral amino acid (AA) uptake. Receptors for numerous hormones, including IGF-I, insulin, and leptin are also present in the MVM (Desoye et al., 1994; Fang et al., 1997; Ebenbichler et al., 2002) suggesting that maternal hormones regulate trophoblast function. Concentrations of maternal serum IGF-I (Holmes et al., 1997) and leptin (Yildiz et al., 2002) are decreased in intrauterine growth restriction (IUGR) while pregnancies associated with obesity and diabetes have higher maternal serum IGF-I, insulin, and leptin (Lauszus et al., 2001; Jansson et al., 2008). This suggests that maternal factors can regulate the activity and expression of transporter proteins in the syncytiotrophoblast, which may influence fetal growth and health.

## Diverse maternal signals impinge on the placenta

A wide range of maternal factors impinges on the placenta, providing critical information about the ability of the maternal supply line to support pregnancy (Jansson et al., 2012; Gaccioli et al., 2013; Jansson and Powell, 2013; Díaz et al., 2014).

### *Utero*-placental blood flow

The development of certain pregnancy complications, particularly IUGR and preeclampsia, is associated with impaired *utero*-placental blood flow. Impaired *utero*-placental blood flow could cause “placental insufficiency”, i.e., impaired nutrient and oxygen supply to the fetus. Placental insufficiency is often assumed to be due only to a reduced placental blood flow (Krishna and Bhalerao, 2011). However, the placental blood flow reduction *per se* does not adequately explain the impaired placental transfer in IUGR. For example, the primary limiting factor for the transplacental transport of nutrients such as glucose and AAs is their transport across the syncytiotrophoblast. We have proposed that the placenta senses the decreased blood flow or possibly hypoxia, and responds by down-regulating key placental nutrient transporters, directly contributing to IUGR. Moreover, the IUGR placenta has reduced intervillous space volume, poorly developed peripheral villi and thicker trophoblastic epithelium that decrease the nutrient exchange area and compromise the exchange functions of the placenta (Burton, 2010).

Animal models of impaired *utero*-placental blood flow show decreased placental nutrient transport capacity. Transplacental transport of glucose and AAs was decreased in IUGR following uterine artery ligation in the rat (Nitzan et al., 1979), however MVM system A activity *in vitro* (Glazier et al., 1996) and placental expression of glucose transporters GLUT 1 and GLUT 3 (Reid et al., 1999) were unaffected. In the guinea pig, IUGR induced by unilateral artery ligation was associated with decreased transplacental AA transport (Jansson and Persson, 1990). In a sheep model of IUGR induced by maternal hyperthermia and decreased *utero*-placental blood flow, transplacental transport of leucine (Ross et al., 1996), threonine (Anderson et al., 1997), glucose (Thureen et al., 1992), and ACP (branched-chain AA analog) (de Vrijer et al., 2004) was reduced.

In human IUGR associated with reduced *utero*-placental blood flow, the activity of several placental AA transporters is reduced whereas placental GLUT1 expression and activity are unaffected (Jansson et al., 1993, 2002b). System A activity is consistently lower in MVM isolated from IUGR placentas (Mahendran et al., 1993; Glazier et al., 1997), especially in preterm IUGR (Jansson et al., 2002b) and is related to the degree of fetal compromise (Glazier et al., 1997). Similarly, the activity of transporters of essential AAs, including system β (taurine) and system L (lysine and leucine), is reduced in MVM and/or BM of IUGR placentas (Jansson et al., 1998; Norberg et al., 1998), consistent with the reduced placental transfer of the essential acids leucine and phenylalanine observed *in vivo* in IUGR pregnancies at term (Paolini et al., 2001). Decreased transplacental AA transport to the fetus may account for the low plasma levels of certain AAs in growth-restricted fetuses (Economides et al., 1989; Cetin et al., 1990). The activity of lipoprotein lipase (LPL), an enzyme responsible for hydrolysis of lipoproteins, is reduced in MVM of IUGR placentas (Magnusson et al., 2004). IUGR is also associated with a reduced placental expression of lipoprotein receptors, low-density lipoprotein (LDL), and scavenger receptor class B type-I, key receptors for cholesterol uptake from maternal LDL and/or HDL (Wadsack et al., 2007). Thus, placental lipid transport may be impaired in IUGR, possibly contributing to the decreased lipid stores in the IUGR fetus (Padoan et al., 2004). Collectively, these data suggest that the effect of reduced *utero*-placental blood flow on fetal growth is mediated, in part, by decreased placental nutrient transfer capacity.

### Hypoxia

Despite compensatory mechanisms such as fetal polycythemia, transplacental transfer of oxygen decreases in maternal hypoxia, which typically is associated with IUGR (Giussani et al., 2007). Women residing at high altitude with reduced oxygen tension have higher risk to deliver IUGR babies than women living at sea level (Zamudio and Moore, 2000; Mehta and Mehta, 2008). Nelson and co-workers reported that hypoxia caused a reduced system A transporter expression and activity in cultured primary human trophoblast cells (Nelson et al., 2003), suggesting that adequate oxygen supply is important for the function of nutrient transporters. Furthermore, high altitude hypoxia decreases the expression of GLUT1 in the syncytiotrophoblast plasma membrane (Zamudio et al., 2006).

### Maternal hormones

Maternal hormones can influence fetal health by altering placental function (Fowden et al., 2015). Maternal IGF-I promotes placental nutrient uptake and transport (Sferruzzi-Perri et al., 2011a). In animal models of IUGR, elevating maternal IGF concentrations improved fetal growth (de Boo et al., 2008). Acute maternal IGF-I treatment in the late pregnant ewe is associated with enhanced glucose delivery to the fetus (Liu et al., 1994). This was also observed in a mouse model of IUGR where placental glucose transporter expression was increased following intraplacental injection of adenovirus-mediated IGF-I (Jones et al., 2013) restoring fetal weights (Keswani et al., 2015). In human trophoblasts, IGF-I increases GLUT1 expression (Baumann et al., 2014) and stimulates glucose and system A-mediated AA uptake (Karl, 1995; Roos et al., 2007). Also, reduced maternal circulating IGF-I is associated with small-for-gestational age and growth-restricted babies (Hernandez-Valencia et al., 2001). IGF-I receptor protein levels were reduced in IUGR (Laviola et al., 2005) and elevated in pregnancies complicated by macrosomia (Jiang et al., 2009).

Insulin and leptin stimulate placental system A activity (Karl et al., 1992; Jansson et al., 2003; von Versen-Hoynck et al., 2009) while adiponectin inhibits insulin-stimulated AA transport (Jones et al., 2010; Rosario et al., 2012; Aye et al., 2014a, 2015). Administration of maternal corticosteroids to pregnant mice during mid-gestation down regulates placental system A transport (Audette et al., 2011) leading to reduced fetal weight (Vaughan et al., 2012). Therefore, maternal hormones influence fetal growth by altering the activity of placental nutrient transporters and placental secretion of hormones (Sferruzzi-Perri et al., 2011a).

### Maternal nutrition

Fetal growth is greatly influenced by maternal nutrition, and is believed to be mediated, in part, by changes in maternal metabolism and hormone levels.

In a rat model of maternal protein restriction, maternal insulin, IGF-I, and leptin levels were decreased (Rosario et al., 2011) and similar changes were observed in a mouse model of calorie restriction (Sferruzzi-Perri et al., 2011b). Maternal corticosterone levels were also increased in this mouse model (Sferruzzi-Perri et al., 2011b). In contrast, pregnant mice on a high fat diet showed increased levels of maternal leptin and decreased adiponectin (Jones et al., 2009b). Consistent with these observations, levels of maternal insulin and leptin were elevated in obese pregnant mice on a high-fat/high-sugar diet (Rosario et al., 2015b).

Maternal endocrine and metabolic changes in response to altered nutrition are similar in experimental models and women. In human IUGR, maternal serum concentrations of IGF-I, insulin, and leptin are decreased (Jansson et al., 2006) while obese pregnant women and pregnancies complicated with gestational diabetes have higher serum levels of leptin, insulin, IGF-I, and decreased levels of adiponectin (Lauszus et al., 2001; Jansson et al., 2008; Aye et al., 2013a, 2015).

Because hormonal regulation of placental nutrient transport is well established, one key mechanism by which maternal nutrition alters placental function and fetal growth could be through modulating placental nutrient transport. Consistent with this hypothesis, various animal experimental models of maternal undernutrition show decreased placental nutrient transport. For example, maternal calorie restriction in the baboon caused IUGR and showed decreased expression and *in vitro* activity of key AA transporters, decreased *in vivo* transplacental AA transport as well as lower fetal levels of essential AAs (Kavitha et al., 2014; Pantham et al., 2015b). Calorie restriction in mice resulted in reduced transplacental glucose and leucine transport (Ganguly et al., 2012). In rats, calorie, or protein restriction in late pregnancy decreased neutral AAs and glucose transplacental transport (Rosso, 1977a,b; Malandro et al., 1996; Jansson et al., 2006; Rosario et al., 2012). Therefore, the proposed cause-and-effect link between maternal undernutrition and decreased fetal growth involves the well-established physiological hormonal response to starvation. Specifically, the increase in the levels of catabolic hormones such as cortisol and the decrease in anabolic hormones including insulin and IGF-I are predicted to inhibit placental nutrient transport, resulting in decreased fetal nutrient availability and IUGR. Opposite placental responses have been reported in maternal over-nutrition in association with fetal overgrowth. In a mouse model of maternal obesity, *in vitro* glucose and AA transporter expression, and activity and *in vivo* transplacental glucose and AA transport are increased (Aye et al., 2015; Rosario et al., 2015b). Importantly, these findings are consistent with up-regulation of placental AA transport in obese women giving birth to large babies (Jansson et al., 2013) and increased placental capacity to transport AAs and glucose in women with diabetes and fetal overgrowth (Jansson et al., 1999, 2002a).

### Inflammatory mediators

Altered inflammatory profile in the mother, placenta, or fetus can affect placental function. Specifically, maternal systemic inflammation has been proposed to play a role in the developmental programming of metabolic disorders especially in pregnancies complicated with obesity and gestational diabetes (Ingvorsen et al., 2015; Pantham et al., 2015a). Male offspring of dams injected with lipopolysaccharide during mid-gestation had enhanced food intake, increased body weight and enlarged abdominal adipose tissue with reduced insulin uptake, consistent with development of obesity and insulin resistance (Nilsson et al., 2001). Offspring of dams exposed to high systemic levels of TNF-α or IL-6 showed increased body weight and adiposity, and exposure to IL-6 alone resulted in insulin resistance in female offspring (Dahlgren et al., 2001).

Maternal obesity in women is associated with a low-grade systemic maternal inflammation and signs of placental inflammation (Challier et al., 2008) However, levels of circulating MCP-1, IL-6, and C-reactive protein that were elevated in early pregnancy in obese women were comparable to those of normal-weight mothers at the end of pregnancy (Ingvorsen et al., 2014) suggesting attenuation of maternal inflammatory state in obese women with advancing gestation. Similarly, women with high BMI had increased circulating levels of MCP-1 and TNF-α and activation of placental inflammatory pathways p38-MAPK and STAT3 (Aye et al., 2014b) without signs of fetal inflammation, suggesting that inflammation associated with maternal overweight/obesity affects the fetus indirectly by modulating placental function.

Maternal circulating cytokines could affect placental function by altering the expression and activity of placental nutrient transporters. IL-6 and TNF-α have been shown to stimulate system A activity in cultured primary human trophoblasts (Jones et al., 2009a). In contrast, IL-1β decreases system A activity in BeWo cells (Thongsong et al., 2005) and inhibits insulin-stimulated system A activity in cultured primary trophoblasts (Aye et al., 2013b). Collectively, these data suggest that a low-grade maternal systemic inflammation in maternal obesity influences fetal growth and programs the fetus for future disease by altering placental functions such as nutrient transport. Whether direct fetal exposure to inflammatory mediators also contributes remains to be established.

#### Placental malaria and IUGR

Every year, ~85 million pregnant women are at risk of malaria, resulting in ~600,000 low birth weight deliveries (Steketee et al., 2001; Desai et al., 2007) mainly attributed to IUGR (Guyatt and Snow, 2004). Recent studies suggest that down-regulation of placental nutrient transport may contribute to IUGR associated with malaria (Boeuf et al., 2013; Chandrasiri et al., 2014).

Placental malaria is the sequestration of *Plasmodium falciparum*-infected erythrocytes in the intervillous space of the placenta (Boeuf et al., 2013). This sequestration can stimulate the recruitment of maternal inflammatory cells such as monocytes and macrophages, a condition termed intervillositis (Ordi et al., 1998) that is associated with an increased risk of low birth weight deliveries (Desai et al., 2007; Rogerson et al., 2007).

Sequestered mononuclear cells and the syncytiotrophoblast can produce various cytokines and chemokines. In placental malaria, intervillous plasma levels of IFN-γ and TNF-α, IL-10, and MCP-1 are increased (Rogerson et al., 2003; Suguitan et al., 2003). Intervillous plasma MIP-1α, IL-8, and monocyte-attracting beta-chemokines such as CCL2 and CCL3 are also increased in placental malaria with intervillositis (Abrams et al., 2003; Bouyou-Akotet et al., 2004; Ioannidis et al., 2014). Inflammation could impair placental development and function, contributing to IUGR in placental malaria. Also, decreased maternal circulating IGF-I levels were observed in women with placental malaria, especially with intervillositis (Umbers et al., 2011). Maternal leptin levels were reduced in mothers with placental malaria (Kabyemela et al., 2008). Importantly, placental malaria with intervillositis is associated with impaired placental AA uptake (Boeuf et al., 2013) and BM GLUT-1 expression (Chandrasiri et al., 2014). Therefore, inflammation, more so than infection, is associated with reduced placental nutrient transport function and deregulation of maternal hormones, which can impact fetal growth and development. The mechanisms underlying the decreased placental nutrient transport capacity and IUGR in placental malaria remain to be fully established.

## mTOR signaling in placental nutrient sensing

Mammalian cells have an array of nutrient-sensing signaling pathways, such as AMP-activated protein kinase (AMPK), AA response signal transduction pathway, glycogen synthase-3 (GSK-3), mTOR, and the hexosamine signaling pathway, which regulate cell metabolism in response to altered nutrient levels. Of these, mTOR is believed to play a central role in placental nutrient sensing (Jansson and Powell, 2006; Jansson et al., 2012). mTOR exists as two protein complexes: mTOR Complex 1 (mTORC1) that regulates cell growth, proliferation, and metabolism and mTORC2 that regulates cytoskeletal organization and cellular metabolism. Placental mTOR signaling likely constitutes a critical link between maternal oxygen and nutrient supply and fetal growth (Jansson et al., 2012).

Hypoxia inhibits mTORC1 signaling by increased expression of DNA damage response 1 (REDD1; Brugarolas et al., 2004) and by activation of AMPK (Inoki et al., 2003). Yung and coworkers also reported that placental mTORC1 signaling is inhibited in women residing at high altitude, consistent with the concept that hypoxia inhibits placental mTORC1 signaling (Yung et al., 2008). In addition, the activity of the placental mTOR signaling pathway is influenced by a multitude of upstream regulators such as amino acids, growth factors, and free fatty acids, which are likely to be affected by maternal nutrition. Protein restriction in rats (Jansson et al., 2006) and nutrient restriction in baboons (Kavitha et al., 2014) resulted in inhibition of placental mTORC1 activity, consistent with human IUGR (Roos et al., 2007; Yung et al., 2008; Chen et al., 2015). In contrast, placental mTOR is activated in animal models of maternal obesity (Jones et al., 2009b; Rosario et al., 2015b) and in obese women delivering large babies (Jansson et al., 2013).

mTOR also has a key role in regulating AA transporters in the human placenta. *In vitro*, mTORC1 positively regulates system A and system L, critical in transplacental AA transport (Rosario et al., 2013). mTORC1 regulates cellular uptake of AAs by affecting the plasma membrane trafficking of transporters by differential ubiquitination, possibly through the ubiquitin ligase, NEDD4-2 (Rosario et al., 2013, 2015a; Chen et al., 2015).

**Figure 2:**
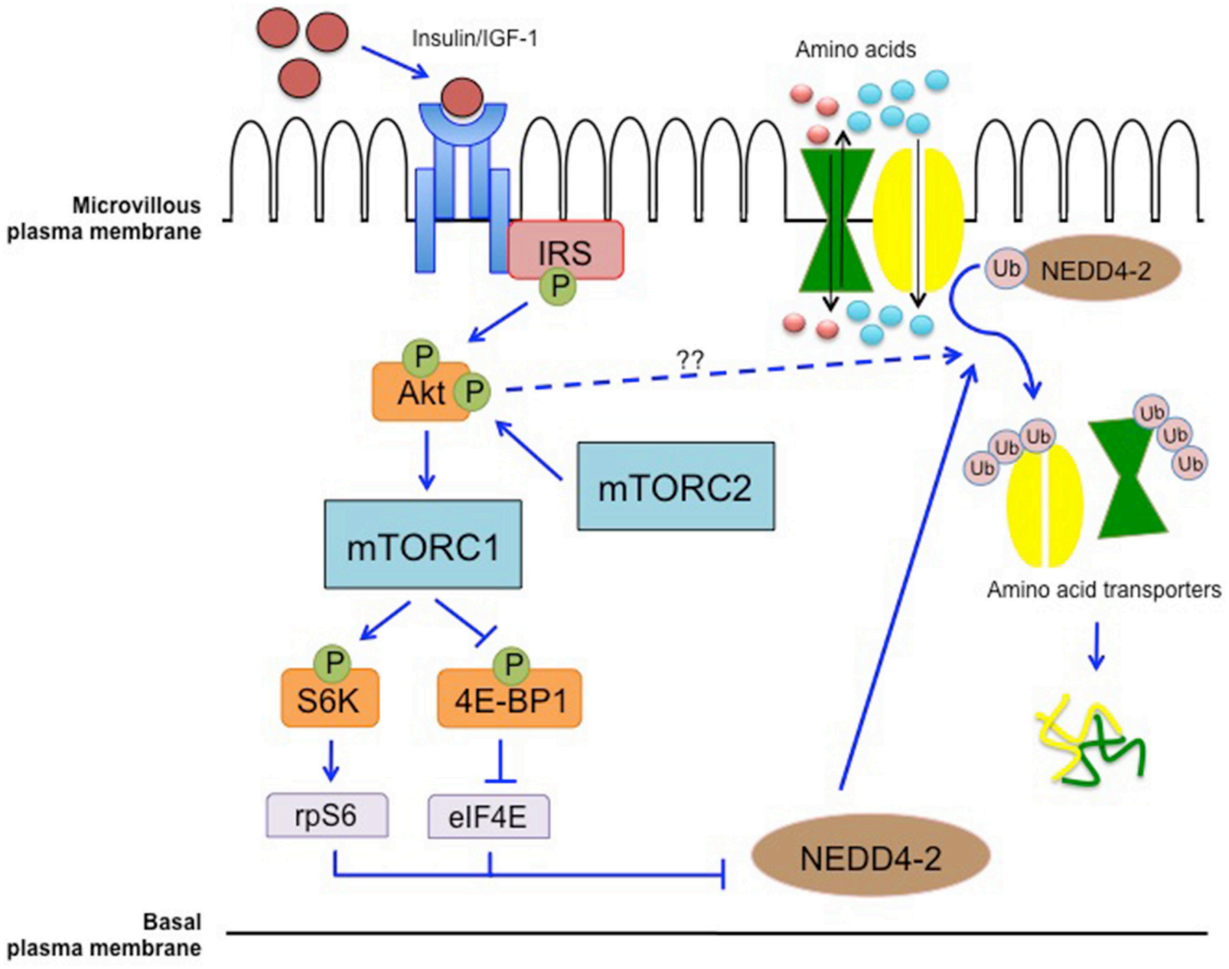
**Placental mTOR as a nutrient sensing signaling pathway**. mTOR integrates maternal signals such as nutrients and growth factors to modulate the transport of nutrients from the maternal to the fetal circulation. mTORC1 modulates the trafficking of AA transporters possibly through differential ubiquitination mediated by NEDD4-2. Inhibition of NEDD4-2 will result in increased localization of AA transporters in the plasma membrane (Rosario et al., 2013, 2015a; Chen et al., 2015).

We propose that mTOR functions as a key placental nutrient sensing signaling pathway responding to upstream maternal signals by modulating transplacental AA transport and influencing the trafficking of nutrient transporters (Figure 2).

## Conclusion and perspectives

Changes in the maternal environment can impair fetal growth and development, which may result in increased susceptibility to diseases in postnatal life. We have proposed that *placental nutrient sensing* allows the placenta to integrate these perturbations with information from intrinsic nutrient sensing signaling pathways to regulate secretion of hormones and placental nutrient and oxygen transfer. Because fetal nutrient supply programs the fetus for future disease, placental function determines the growth and life-long health of the fetus.

Placental responses to perturbations in the maternal compartment are complex and remain poorly understood, highlighting an urgent need for further well-designed and mechanistic research in this area. Intervention strategies to alleviate pregnancy complications and prevent fetal programming of adult disease are likely to be most effective if placental function is targeted. mTOR constitutes an important nutrient sensing signaling pathway believed to play a key role in placental nutrient sensing. Maternal obesity with fetal overgrowth is associated with activation of placental mTOR signaling and up-regulation of placental nutrient transport both in animal models (Aye et al., 2015; Rosario et al., 2015a,b) and in women (Jansson et al., 2013). We recently reported that normalization of maternal circulating levels of adiponectin in obese mice completely prevented the activation of placental mTOR signaling, up-regulation of placental nutrient transport and fetal overgrowth (Aye et al., 2015) consistent with the idea that targeting placental mTOR may represent an effective intervention strategy in cases of abnormal fetal growth.

## Author contributions

KD and TJ wrote the paper. PB and TP edited the paper.

## Funding

This work was funded by NIH grants HD078376, HD065007, HD68370, R24OD016724, HD021350, and DK089989.

### Conflict of interest statement

The authors declare that the research was conducted in the absence of any commercial or financial relationships that could be construed as a potential conflict of interest.

## References

[B1] Abrams E. T. Brown H. Chensue S. W. Turner G. D. Tadesse E. Lema V. M.. (2003). Host response to malaria during pregnancy: placental monocyte recruitment is associated with elevated beta chemokine expression. J. Immunol. 170, 2759–2764. 10.4049/jimmunol.170.5.2759 12594307

[B2] Anderson A. H. Fennessey P. V. Meschia G. Wilkening R. B. Battaglia F. C. (1997). Placental transport of threonine and its utilization in the normal and growth-restricted fetus. Am. J. Physiol. 272, E892–E900. 9176191 10.1152/ajpendo.1997.272.5.E892

[B3] Armitage J. A. Khan I. Y. Taylor P. D. Nathanielsz P. W. Poston L. (2004). Developmental programming of the metabolic syndrome by maternal nutritional imbalance: how strong is the evidence from experimental models in mammals? J. Physiol. 561, 355–377. 10.1113/jphysiol.2004.072009 15459241 PMC1665360

[B4] Audette M. C. Challis J. R. Jones R. L. Sibley C. P. Matthews S. G. (2011). Antenatal dexamethasone treatment in midgestation reduces system A-mediated transport in the late-gestation murine placenta. Endocrinology 152, 3561–3570. 10.1210/en.2011-0104 21733830

[B5] Aye I. L. Gao X. Weintraub S. T. Jansson T. Powell T. L. (2014a). Adiponectin inhibits insulin function in primary trophoblasts by PPARalpha-mediated ceramide synthesis. Mol. Endocrinol. 28, 512–524. 10.1210/me.2013-1401 24606127 PMC3968401

[B6] Aye I. L. Lager S. Ramirez V. I. Gaccioli F. Dudley D. J. Jansson T.. (2014b). Increasing maternal body mass index is associated with systemic inflammation in the mother and the activation of distinct placental inflammatory pathways. Biol. Reprod. 90, 129. 10.1095/biolreprod.113.116186 24759787 PMC4094003

[B7] Aye I. L. M. H. Jansson T. Powell T. L. (2013b). Interleukin-1 beta inhibits insulin signaling and prevents insulin-stimulated system A amino acid transport in primary human trophoblasts. Mol. Cell. Endocrinol. 381, 46–55. 10.1016/j.mce.2013.07.013 23891856 PMC3795822

[B8] Aye I. L. Powell T. L. Jansson T. (2013a). Review: adiponectin–the missing link between maternal adiposity, placental transport and fetal growth? Placenta 34 Suppl., S40–S45. 10.1016/j.placenta.2012.11.024 23245987 PMC3650089

[B9] Aye I. L. Rosario F. J. Powell T. L. Jansson T. (2015). Adiponectin supplementation in pregnant mice prevents the adverse effects of maternal obesity on placental function and fetal growth. Proc. Natl. Acad. Sci. U.S.A. 112, 12858–12863. 10.1073/pnas.1515484112 26417088 PMC4611638

[B10] Barker D. J. Osmond C. Golding J. Kuh D. Wadsworth M. E. (1989). Growth in *utero*, blood pressure in childhood and adult life, and mortality from cardiovascular disease. BMJ 298, 564–567. 10.1136/bmj.298.6673.564 2495113 PMC1835925

[B11] Barker D. J. P. Osmond C. (1986). Infant-mortality, childhood nutrition, and ischemic-heart-disease in England and Wales. Lancet 1, 1077–1081. 10.1016/S0140-6736(86)91340-1 2871345

[B12] Baumann M. U. Schneider H. Malek A. Palta V. Surbek D. V. Sager R.. (2014). Regulation of human trophoblast GLUT1 glucose transporter by insulin-like growth factor I (IGF-I). PLoS ONE 9:e106037. 10.1371/journal.pone.0106037 25157747 PMC4144961

[B13] Boeuf P. Aitken E. H. Chandrasiri U. Chua C. L. McInerney B. McQuade L.. (2013). *Plasmodium falciparum* malaria elicits inflammatory responses that dysregulate placental amino acid transport. PLoS Pathog. 9:e1003153. 10.1371/journal.ppat.1003153 23408887 PMC3567154

[B14] Bouyou-Akotet M. K. Kombila M. Kremsner P. G. Mavoungou E. (2004). Cytokine profiles in peripheral, placental and cord blood in pregnant women from an area endemic for *Plasmodium falciparum*. Eur. Cytokine Netw. 15, 120–125. 15319171

[B15] Brugarolas J. Lei K. Hurley R. L. Manning B. D. Reiling J. H. Hafen E.. (2004). Regulation of mTOR function in response to hypoxia by REDD1 and the TSC1/TSC2 tumor suppressor complex. Genes Dev. 18, 2893–2904. 10.1101/gad.1256804 15545625 PMC534650

[B16] Burton G. J. (2010). The influence of the intrauterine environment on human placental development. J. Reprod. Immunol. 86, 81–82. 10.1016/j.jri.2010.08.006 19757391

[B17] Cetin I. Corbetta C. Sereni L. P. Marconi A. M. Bozzetti P. Pardi G.. (1990). Umbilical amino acid concentrations in normal and growth-retarded fetuses sampled in *utero* by cordocentesis. Am. J. Obstet. Gynecol. 162, 253–261. 10.1016/0002-9378(90)90860-A 2301500

[B18] Challier J. C. Basu S. Bintein T. Minium J. Hotmire K. Catalano P. M.. (2008). Obesity in pregnancy stimulates macrophage accumulation and inflammation in the placenta. Placenta 29, 274–281. 10.1016/j.placenta.2007.12.010 18262644 PMC4284075

[B19] Chandrasiri U. P. Chua C. L. Umbers A. J. Chaluluka E. Glazier J. D. Rogerson S. J.. (2014). Insight into the pathogenesis of fetal growth restriction in placental malaria: decreased placental glucose transporter isoform 1 expression. J. Infect. Dis. 209, 1663–1667. 10.1093/infdis/jit803 24325968

[B20] Chen Y. Y. Rosario F. J. Shehab M. A. Powell T. L. Gupta M. B. Jansson T. (2015). Increased ubiquitination and reduced plasma membrane trafficking of placental amino acid transporter SNAT-2 in human IUGR. Clin. Sci. 129, 1131–1141. 10.1042/CS20150511 26374858 PMC4614027

[B21] Dahlgren J. Nilsson C. Jennische E. Ho H. P. Eriksson E. Niklasson A.. (2001). Prenatal cytokine exposure results in obesity and gender-specific programming. Am. J. Physiol. Endocrinol. Metab. 281, E326–E334. 11440909 10.1152/ajpendo.2001.281.2.E326

[B22] de Boo H. A. Eremia S. C. Bloomfield F. H. Oliver M. H. Harding J. E. (2008). Treatment of intrauterine growth restriction with maternal growth hormone supplementation in sheep. Am. J. Obstet. Gynecol. 199, e551–e559. 10.1016/j.ajog.2008.04.035 18599015

[B23] Desai M. ter Kuile F. O. Nosten F. McGready R. Asamoa K. Brabin B.. (2007). Epidemiology and burden of malaria in pregnancy. Lancet Infect. Dis. 7, 93–104. 10.1016/S1473-3099(07)70021-X 17251080

[B24] Desoye G. Hartmann M. Blaschitz A. Dohr G. Hahn T. Kohnen G.. (1994). Insulin receptors in syncytiotrophoblast and fetal endothelium of human placenta. Immunohistochemical evidence for developmental changes in distribution pattern. Histochemistry 101, 277–285. 10.1007/BF00315915 7928411

[B25] de Vrijer B. Regnault T. R. Wilkening R. B. Meschia G. Battaglia F. C. (2004). Placental uptake and transport of ACP, a neutral nonmetabolizable amino acid, in an ovine model of fetal growth restriction. Am. J. Physiol. Endocrinol. Metab. 287, E1114–E1124. 10.1152/ajpendo.00259.2004 15315907

[B26] Díaz P. Powell T. L. Jansson T. (2014). The role of placental nutrient sensing in maternal-fetal resource allocation. Biol. Reprod. 91, 82. 10.1095/biolreprod.114.121798 25122064 PMC4435028

[B27] Ebenbichler C. F. Kaser S. Laimer M. Wolf H. J. Patsch J. R. Illsley N. P. (2002). Polar expression and phosphorylation of human leptin receptor isoforms in paired, syncytial, microvillous and basal membranes from human term placenta. Placenta 23, 516–521. 10.1053/plac.2002.0836 12137750

[B28] Economides D. L. Nicolaides K. H. Gahl W. A. Bernardini I. Evans M. I. (1989). Plasma amino acids in appropriate- and small-for-gestational-age fetuses. Am. J. Obstet. Gynecol. 161, 1219–1227. 10.1016/0002-9378(89)90670-4 2589443

[B29] Fang J. Furesz T. C. Lurent R. S. Smith C. H. Fant M. E. (1997). Spatial polarization of insulin-like growth factor receptors on the human syncytiotrophoblast. Pediatr. Res. 41, 258–265. 10.1203/00006450-199702000-00017 9029648

[B30] Forsdahl A. (1977). Are poor living conditions in childhood and adolescence an important risk factor for arteriosclerotic heart disease? Br. J. Prev. Soc. Med. 31, 91–95. 10.1136/jech.31.2.91 884401 PMC479002

[B31] Fowden A. L. Forhead A. J. Sferruzzi-Perri A. N. Burton G. J. Vaughan O. R. (2015). Review: endocrine regulation of placental phenotype. Placenta 36(Suppl. 1), S50–S59. 10.1016/j.placenta.2014.11.018 25524059

[B32] Gaccioli F. Lager S. Powell T. L. Jansson T. (2013). Placental transport in response to altered maternal nutrition. J. Dev. Orig. Health Dis. 4, 101–115. 10.1017/S2040174412000529 25054676 PMC4237017

[B33] Ganguly A. Collis L. Devaskar S. U. (2012). Placental glucose and amino acid transport in calorie-restricted wild-type and Glut3 null heterozygous mice. Endocrinology 153, 3995–4007. 10.1210/en.2011-1973 22700768 PMC3404359

[B34] Giussani D. A. Salinas C. E. Villena M. Blanco C. E. (2007). The role of oxygen in prenatal growth: studies in the chick embryo. J. Physiol. 585, 911–917. 10.1113/jphysiol.2007.141572 17962335 PMC2375513

[B35] Glazier J. D. Cetin I. Perugino G. Ronzoni S. Grey A. M. Mahendran D.. (1997). Association between the activity of the system A amino acid transporter in the microvillous plasma membrane of the human placenta and severity of fetal compromise in intrauterine growth restriction. Pediatr. Res. 42, 514–519. 10.1203/00006450-199710000-00016 9380446

[B36] Glazier J. D. Sibley C. P. Carter A. M. (1996). Effect of fetal growth restriction on system A amino acid transporter activity in the maternal facing plasma membrane of rat syncytiotrophoblast. Pediatr. Res. 40, 325–329. 10.1203/00006450-199608000-00022 8827785

[B37] Gluckman P. D. Hanson M. A. (2004). Living with the past: evolution, development, and patterns of disease. Science 305, 1733–1736. 10.1126/science.1095292 15375258

[B38] Guyatt H. L. Snow R. W. (2004). Impact of malaria during pregnancy on low birth weight in sub-Saharan Africa. Clin. Microbiol. Rev. 17, 760–769. 10.1128/CMR.17.4.760-769.2004 15489346 PMC523568

[B39] Hales C. N. Barker D. J. P. Clark P. M. S. Cox L. J. Fall C. Osmond C.. (1991). Fetal and infant growth and impaired glucose-tolerance at age 64. Br. Med. J. 303, 1019–1022. 10.1136/bmj.303.6809.1019 1954451 PMC1671766

[B40] Hernandez-Valencia M. Zarate A. Ochoa R. Fonseca M. E. Amato D. De Jesus Ortiz M. (2001). Insulin-like growth factor I, epidermal growth factor and transforming growth factor beta expression and their association with intrauterine fetal growth retardation, such as development during human pregnancy. Diabetes Obes. Metab. 3, 457–462. 10.1046/j.1463-1326.2001.00168.x 11903419

[B41] Holmes R. Montemagno R. Jones J. Preece M. Rodeck C. Soothill P. (1997). Fetal and maternal plasma insulin-like growth factors and binding proteins in pregnancies with appropriate or retarded fetal growth. Early Hum. Dev. 49, 7–17. 10.1016/S0378-3782(97)01867-7 9179534

[B42] Ingvorsen C. Brix S. Ozanne S. E. Hellgren L. I. (2015). The effect of maternal inflammation on foetal programming of metabolic disease. Acta Physiol. 214, 440–449. 10.1111/apha.12533 26011013

[B43] Ingvorsen C. Thysen A. H. Fernandez-Twinn D. Nordby P. Nielsen K. F. Ozanne S. E.. (2014). Effects of pregnancy on obesity-induced inflammation in a mouse model of fetal programming. Int. J. Obes. 38, 1282–1289. 10.1038/ijo.2014.69 24785102

[B44] Inoki K. Zhu T. Guan K. L. (2003). TSC2 mediates cellular energy response to control cell growth and survival. Cell 115, 577–590. 10.1016/S0092-8674(03)00929-2 14651849

[B45] Ioannidis L. J. Nie C. Q. Hansen D. S. (2014). The role of chemokines in severe malaria: more than meets the eye. Parasitology 141, 602–613. 10.1017/S0031182013001984 24476686 PMC3962270

[B46] Jansson N. Greenwood S. L. Johansson B. R. Powell T. L. Jansson T. (2003). Leptin stimulates the activity of the system A amino acid transporter in human placental villous fragments. J. Clin. Endocrinol. Metab. 88, 1205–1211. 10.1210/jc.2002-021332 12629107

[B47] Jansson N. Nilsfelt A. Gellerstedt M. Wennergren M. Rossander-Hulthén L. Powell T. L.. (2008). Maternal hormones linking maternal body mass index and dietary intake to birth weight. Am. J. Clin. Nutr. 87, 1743–1749. 18541564 10.1093/ajcn/87.6.1743

[B48] Jansson N. Pettersson J. Haafiz A. Ericsson A. Palmberg I. Tranberg M.. (2006). Down-regulation of placental transport of amino acids precedes the development of intrauterine growth restriction in rats fed a low protein diet. J. Physiol. Lond. 576, 935–946. 10.1113/jphysiol.2003.550004 16916910 PMC1892642

[B49] Jansson N. Rosario F. J. Gaccioli F. Lager S. Jones H. N. Roos S.. (2013). Activation of placental mTOR signaling and amino acid transporters in obese women giving birth to large babies. J. Clin. Endocrinol. Metab. 98, 105–113. 10.1210/jc.2012-2667 23150676 PMC3537112

[B50] Jansson T. Aye I. L. Goberdhan D. C. (2012). The emerging role of mTORC1 signaling in placental nutrient-sensing. Placenta 33(Suppl. 2), e23–e29. 10.1016/j.placenta.2012.05.010 22687819 PMC3463762

[B51] Jansson T. Ekstrand Y. Björn C. Wennergren M. Powell T. L. (2002a). Alterations in the activity of placental amino acid transporters in pregnancies complicated by diabetes. Diabetes 51, 2214–2219. 10.2337/diabetes.51.7.2214 12086952

[B52] Jansson T. Persson E. (1990). Placental-transfer of glucose and amino-acids in intrauterine growth-retardation – studies with substrate-analogs in the awake guinea-pig. Pediatr. Res. 28, 203–208. 10.1203/00006450-199009000-00007 2235115

[B53] Jansson T. Powell T. L. (2006). Human placental transport in altered fetal growth: does the placenta function as a nutrient sensor? Rev. Placenta 27, S91–S97. 10.1016/j.placenta.2005.11.010 16442615

[B54] Jansson T. Powell T. L. (2013). Role of placental nutrient sensing in developmental programming. Clin. Obstet. Gynecol. 56, 591–601. 10.1097/GRF.0b013e3182993a2e 23703224 PMC3732521

[B55] Jansson T. Scholtbach V. Powell T. L. (1998). Placental transport of leucine and lysine is reduced in intrauterine growth restriction. Pediatr. Res. 44, 532–537. 10.1203/00006450-199810000-00011 9773842

[B56] Jansson T. Wennergren M. Illsley N. P. (1993). Glucose transporter protein expression in human placenta throughout gestation and in intrauterine growth retardation. J. Clin. Endocrinol. Metab. 77, 1554–1562. 8263141 10.1210/jcem.77.6.8263141

[B57] Jansson T. Wennergren M. Powell T. L. (1999). Placental glucose transport and GLUT 1 expression in insulin-dependent diabetes. Am. J. Obstet. Gynecol. 180, 163–168. 10.1016/S0002-9378(99)70169-9 9914598

[B58] Jansson T. Ylvén K. Wennergren M. Powell T. L. (2002b). Glucose transport and system A activity in syncytiotrophoblast microvillous and basal plasma membranes in intrauterine growth restriction. Placenta 23, 392–399. 10.1053/plac.2002.0826 12061855

[B59] Jiang H. Xun P. C. Luo G. H. Wang Q. W. Cai Y. Q. Zhang Y. Y.. (2009). Levels of insulin-like growth factors and their receptors in placenta in relation to macrosomia. Asia Pac. J. Clin. Nutr. 18, 171–178. 19713175

[B60] Jones H. N. Crombleholme T. Habli M. (2013). Adenoviral-mediated placental gene transfer of IGF-1 corrects placental insufficiency via enhanced placental glucose transport mechanisms. PLoS ONE 8:e74632. 10.1371/journal.pone.0074632 24019972 PMC3760855

[B61] Jones H. N. Jansson T. Powell T. L. (2009a). IL-6 stimulates system A amino acid transporter activity in trophoblast cells through STAT3 and increased expression of SNAT2. Am. J. Physiol. Cell Physiol. 297, C1228–C1235. 10.1152/ajpcell.00195.2009 19741197

[B62] Jones H. N. Jansson T. Powell T. L. (2010). Full-length adiponectin attenuates insulin signaling and inhibits insulin-stimulated amino Acid transport in human primary trophoblast cells. Diabetes 59, 1161–1170. 10.2337/db09-0824 20150288 PMC2857896

[B63] Jones H. N. Powell T. L. Jansson T. (2007). Regulation of placental nutrient transport–a review. Placenta 28, 763–774. 10.1016/j.placenta.2007.05.002 17582493

[B64] Jones H. N. Woollett L. A. Barbour N. Prasad P. D. Powell T. L. Jansson T. (2009b). High-fat diet before and during pregnancy causes marked up-regulation of placental nutrient transport and fetal overgrowth in C57/BL6 mice. FASEB J. 23, 271–278. 10.1096/fj.08-116889 18827021 PMC2626621

[B65] Kabyemela E. R. Muehlenbachs A. Fried M. Kurtis J. D. Mutabingwa T. K. Duffy P. E. (2008). Maternal peripheral blood level of IL-10 as a marker for inflammatory placental malaria. Malar. J. 7:26. 10.1186/1475-2875-7-26 18230163 PMC2265723

[B66] Karl P. I. (1995). Insulin-like growth factor-1 stimulates amino acid uptake by the cultured human placental trophoblast. J. Cell. Physiol. 165, 83–88. 10.1002/jcp.1041650111 7559811

[B67] Karl P. I. Alpy K. L. Fisher S. E. (1992). Amino acid transport by the cultured human placental trophoblast: effect of insulin on AIB transport. Am. J. Physiol. 262, C834–C839. 1566812 10.1152/ajpcell.1992.262.4.C834

[B68] Kavitha J. V. Rosario F. J. Nijland M. J. McDonald T. J. Wu G. Kanai Y.. (2014). Down-regulation of placental mTOR, insulin/IGF-I signaling, and nutrient transporters in response to maternal nutrient restriction in the baboon. FASEB J. 28, 1294–1305. 10.1096/fj.13-242271 24334703 PMC3929672

[B69] Keswani S. G. Balaji S. Katz A. B. King A. Omar K. Habli M.. (2015). Intraplacental gene therapy with Ad-IGF-1 corrects naturally occurring rabbit model of intrauterine growth restriction. Hum. Gene Ther. 26, 172–182. 10.1089/hum.2014.065 25738403 PMC4367241

[B70] Krishna U. Bhalerao S. (2011). Placental insufficiency and fetal growth restriction. J. Obstet. Gynaecol. India 61, 505–511. 10.1007/s13224-011-0092-x 23024517 PMC3257343

[B71] Lauszus F. F. Klebe J. G. Flyvbjerg A. (2001). Macrosomia associated with maternal serum insulin-like growth factor-I and -II in diabetic pregnancy. Obstet. Gynecol. 97, 734–741. 10.1016/S0029-7844(01)01189-9 11339926

[B72] Laviola L. Perrini S. Belsanti G. Natalicchio A. Montrone C. Leonardini A.. (2005). Intrauterine growth restriction in humans is associated with abnormalities in placental insulin-like growth factor signaling. Endocrinology 146, 1498–1505. 10.1210/en.2004-1332 15564321

[B73] Liu L. Harding J. E. Evans P. C. Gluckman P. D. (1994). Maternal insulin-like growth factor-I infusion alters feto-placental carbohydrate and protein metabolism in pregnant sheep. Endocrinology 135, 895–900. 8070384 10.1210/endo.135.3.8070384

[B74] Magnusson A. L. Waterman I. J. Wennergren M. Jansson T. Powell T. L. (2004). Triglyceride hydrolase activities and expression of fatty acid binding proteins in the human placenta in pregnancies complicated by intrauterine growth restriction and diabetes. J. Clin. Endocrinol. Metab. 89, 4607–4614. 10.1210/jc.2003-032234 15356070

[B75] Mahendran D. Donnai P. Glazier J. D. D'souza S. W. Boyd R. D. Sibley C. P. (1993). Amino acid (system A) transporter activity in microvillous membrane vesicles from the placentas of appropriate and small for gestational age babies. Pediatr. Res. 34, 661–665. 10.1203/00006450-199311000-00019 8284106

[B76] Malandro M. S. Beveridge M. J. Kilberg M. S. Novak D. A. (1996). Effect of low-protein diet-induced intrauterine growth retardation on rat placental amino acid transport. Am. J. Physiol. 271, C295–C303. 8760058 10.1152/ajpcell.1996.271.1.C295

[B77] Mehta A. R. Mehta P. R. (2008). The hypoxia of high altitude causes restricted fetal growth in chick embryos with the extent of this effect depending on maternal altitudinal status. J. Physiol. 586, 1469–1471. 10.1113/jphysiol.2008.151332 18238814 PMC2375702

[B78] Nelson D. M. Smith S. D. Furesz T. C. Sadovsky Y. Ganapathy V. Parvin C. A.. (2003). Hypoxia reduces expression and function of system A amino acid transporters in cultured term human trophoblasts. Am. J. Physiol. Cell Physiol. 284, C310–C315. 10.1152/ajpcell.00253.2002 12388062

[B79] Nilsson C. Larsson B. M. Jennische E. Eriksson E. Björntorp P. York D. A.. (2001). Maternal endotoxemia results in obesity and insulin resistance in adult male offspring. Endocrinology 142, 2622–2630. 10.1210/en.142.6.2622 11356713

[B80] Nitzan M. Orloff S. Schulman J. D. (1979). Placental transfer of analogs of glucose and amino acids in experimental intrauterine growth retardation. Pediatr. Res. 13, 100–103. 10.1203/00006450-197902000-00003 432004

[B81] Norberg S. Powell T. L. Jansson T. (1998). Intrauterine growth restriction is associated with a reduced activity of placental taurine transporters. Pediatr. Res. 44, 233–238. 10.1203/00006450-199808000-00016 9702920

[B82] Ordi J. Ismail M. R. Ventura P. J. Kahigwa E. Hirt R. Cardesa A.. (1998). Massive chronic intervillositis of the placenta associated with malaria infection. Am. J. Surg. Pathol. 22, 1006–1011. 10.1097/00000478-199808000-00011 9706981

[B83] Padoan A. Rigano S. Ferrazzi E. Beaty B. L. Battaglia F. C. Galan H. L. (2004). Differences in fat and lean mass proportions in normal and growth-restricted fetuses. Am. J. Obstet. Gynecol. 191, 1459–1464. 10.1016/j.ajog.2004.06.045 15507983

[B84] Pantham P. Aye I. L. Powell T. L. (2015a). Inflammation in maternal obesity and gestational diabetes mellitus. Placenta 36, 709–715. 10.1016/j.placenta.2015.04.006 25972077 PMC4466145

[B85] Pantham P. Rosario F. J. Njiland M. Cheung A. Nathanielsz P. W. Powell T. L.. (2015b). Reduced placental amino acid transport in response to maternal nutrient restriction in the baboon. Am. J. Physiol. Regul. Integr. Comp. Physiol. 309, R740–R746. 10.1152/ajpregu.00161.2015 26246504 PMC4666932

[B86] Paolini C. L. Marconi A. M. Ronzoni S. Di Noio M. Fennessey P. V. Pardi G.. (2001). Placental transport of leucine, phenylalanine, glycine, and proline in intrauterine growth-restricted pregnancies. J. Clin. Endocrinol. Metab. 86, 5427–5432. 10.1210/jcem.86.11.8036 11701717

[B87] Ravelli A. C. J. van der Meulen J. H. P. Michels R. P. J. Osmond C. Barker D. J. P. Hales C. N.. (1998). Glucose tolerance in adults after prenatal exposure to famine. Lancet 351, 173–177. 10.1016/S0140-6736(97)07244-9 9449872

[B88] Reid G. J. Lane R. H. Flozak A. S. Simmons R. A. (1999). Placental expression of glucose transporter proteins 1 and 3 in growth-restricted fetal rats. Am. J. Obstet. Gynecol. 180, 1017–1023. 10.1016/S0002-9378(99)70675-7 10203672

[B89] Rogerson S. J. Brown H. C. Pollina E. Abrams E. T. Tadesse E. Lema V. M.. (2003). Placental tumor necrosis factor alpha but not gamma interferon is associated with placental malaria and low birth weight in Malawian women. Infect. Immun. 71, 267–270. 10.1128/IAI.71.1.267-270.2003 12496175 PMC143363

[B90] Rogerson S. J. Hviid L. Duffy P. E. Leke R. F. Taylor D. W. (2007). Malaria in pregnancy: pathogenesis and immunity. Lancet Infect. Dis. 7, 105–117. 10.1016/S1473-3099(07)70022-1 17251081

[B91] Roos S. Jansson N. Palmberg I. Säljö K. Powell T. L. Jansson T. (2007). Mammalian target of rapamycin in the human placenta regulates leucine transport and is down-regulated in restricted fetal growth. J. Physiol. 582, 449–459. 10.1113/jphysiol.2007.129676 17463046 PMC2075295

[B92] Roos S. Kanai Y. Prasad P. D. Powell T. L. Jansson T. (2009). Regulation of placental amino acid transporter activity by mammalian target of rapamycin. Am. J. Physiol. Cell Physiol. 296, C142–C150. 10.1152/ajpcell.00330.2008 18987252

[B93] Rosario F. J. Dimasuay K. G. Kanai Y. Powell T. L. Jansson T. (2015a). Regulation of amino acid transporter trafficking by mTORC1 in primary human trophoblast cells is mediated by the ubiquitin ligase Nedd4-2. Clin Sci (Lond). [Epub ahead of print]. 10.1042/CS20150554. 26608079 PMC5681479

[B94] Rosario F. J. Jansson N. Kanai Y. Prasad P. D. Powell T. L. Jansson T. (2011). Maternal protein restriction in the rat inhibits placental insulin, mTOR, and STAT3 signaling and down-regulates placental amino acid transporters. Endocrinology 152, 1119–1129. 10.1210/en.2010-1153 21285325 PMC3858644

[B95] Rosario F. J. Kanai Y. Powell T. L. Jansson T. (2013). Mammalian target of rapamycin signalling modulates amino acid uptake by regulating transporter cell surface abundance in primary human trophoblast cells. J. Physiol. 591, 609–625. 10.1113/jphysiol.2012.238014 23165769 PMC3577540

[B96] Rosario F. J. Kanai Y. Powell T. L. Jansson T. (2015b). Increased placental nutrient transport in a novel mouse model of maternal obesity with fetal overgrowth. Obesity 23, 1663–1670. 10.1002/oby.21165 26193061 PMC4509489

[B97] Rosario F. J. Schumacher M. A. Jiang J. Kanai Y. Powell T. L. Jansson T. (2012). Chronic maternal infusion of full-length adiponectin in pregnant mice down-regulates placental amino acid transporter activity and expression and decreases fetal growth. J. Physiol. (Lond). 590, 1495–1509. 10.1113/jphysiol.2011.226399 22289908 PMC3382336

[B98] Ross J. C. Fennessey P. V. Wilkening R. B. Battaglia F. C. Meschia G. (1996). Placental transport and fetal utilization of leucine in a model of fetal growth retardation. Am. J. Physiol. 270, E491–E503. 8638698 10.1152/ajpendo.1996.270.3.E491

[B99] Rosso P. (1977a). Maternal-fetal exchange during protein malnutrition in the rat. Placental transfer of alpha-amino isobutyric acid. J. Nutr. 107, 2002–2005. 908958 10.1093/jn/107.11.2002

[B100] Rosso P. (1977b). Maternal-fetal exchange during protein malnutrition in the rat. Placental transfer of glucose and a nonmetabolizable glucose analog. J. Nutr. 107, 20006–20010. 908957 10.1093/jn/107.11.2006

[B101] Sferruzzi-Perri A. N. Owens J. A. Pringle K. G. Roberts C. T. (2011a). The neglected role of insulin-like growth factors in the maternal circulation regulating fetal growth. J. Physiol. (Lond). 589, 7–20. 10.1113/jphysiol.2010.198622 20921199 PMC3021777

[B102] Sferruzzi-Perri A. N. Vaughan O. R. Coan P. M. Suciu M. C. Darbyshire R. Constancia M.. (2011b). Placental-specific Igf2 deficiency alters developmental adaptations to undernutrition in mice. Endocrinology 152, 3202–3212. 10.1210/en.2011-0240 21673101

[B103] Steketee R. W. Nahlen B. L. Parise M. E. Menendez C. (2001). The burden of malaria in pregnancy in malaria-endemic areas. Am. J. Trop. Med. Hyg. 64, 28–35. 11425175 10.4269/ajtmh.2001.64.28

[B104] Suguitan A. L. Jr. Leke R. G. Fouda G. Zhou A. Thuita L. Metenou S.. (2003). Changes in the levels of chemokines and cytokines in the placentas of women with *Plasmodium falciparum* malaria. J. Infect. Dis. 188, 1074–1082. 10.1086/378500 14513430

[B105] Thongsong B. Subramanian R. K. Ganapathy V. Prasad P. D. (2005). Inhibition of amino acid transport system a by interleukin-1 beta in trophoblasts. J. Soc. Gynecol. Investig. 12, 495–503. 10.1016/j.jsgi.2005.06.008 16202926

[B106] Thureen P. J. Trembler K. A. Meschia G. Makowski E. L. Wilkening R. B. (1992). Placental glucose transport in heat-induced fetal growth retardation. Am. J. Physiol. 263, R578–R585. 1415644 10.1152/ajpregu.1992.263.3.R578

[B107] Umbers A. J. Boeuf P. Clapham C. Stanisic D. I. Baiwog F. Mueller I.. (2011). Placental malaria-associated inflammation disturbs the insulin-like growth factor axis of fetal growth regulation. J. Infect. Dis. 203, 561–569. 10.1093/infdis/jiq080 21216864 PMC3071224

[B108] Vaughan O. R. Sferruzzi-Perri A. N. Fowden A. L. (2012). Maternal corticosterone regulates nutrient allocation to fetal growth in mice. J. Physiol. (Lond). 590, 5529–5540. 10.1113/jphysiol.2012.239426 22930269 PMC3515836

[B109] von Versen-Höynck F. Rajakumar A. Parrott M. S. Powers R. W. (2009). Leptin affects system A amino acid transport activity in the human placenta: evidence for STAT3 dependent mechanisms. Placenta 30, 361–367. 10.1016/j.placenta.2009.01.004 19203792 PMC2675556

[B110] Wadsack C. Tabano S. Maier A. Hiden U. Alvino G. Cozzi V.. (2007). Intrauterine growth restriction is associated with alterations in placental lipoprotein receptors and maternal lipoprotein composition. Am. J. Physiol. Endocrinol. Metab. 292, E476–E484. 10.1152/ajpendo.00547.2005 17003234

[B111] Yildiz L. Avci B. Ingeç M. (2002). Umbilical cord and maternal blood leptin concentrations in intrauterine growth retardation. Clin. Chem. Lab. Med. 40, 1114–1117. 10.1515/cclm.2002.195 12521228

[B112] Yung H. W. Calabrese S. Hynx D. Hemmings B. A. Cetin I. Charnock-Jones D. S.. (2008). Evidence of placental translation inhibition and endoplasmic reticulum stress in the etiology of human intrauterine growth restriction. Am. J. Pathol. 173, 451–462. 10.2353/ajpath.2008.071193 18583310 PMC2475782

[B113] Zamudio S. Baumann M. U. Illsley N. P. (2006). Effects of chronic hypoxia *in vivo* on the expression of human placental glucose transporters. Placenta 27, 49–55. 10.1016/j.placenta.2004.12.010 16310037 PMC4497571

[B114] Zamudio S. Moore L. G. (2000). Altitude and fetal growth: current knowledge and future directions. Ultrasound Obstet. Gynecol. 16, 6–8. 10.1046/j.1469-0705.2000.00155.x 11084958

